# Gene and environmental interactions according to the components of lifestyle modifications in hypertension guidelines

**DOI:** 10.1186/s12199-019-0771-2

**Published:** 2019-03-11

**Authors:** Yoshihiro Kokubo, Sandosh Padmanabhan, Yoshio Iwashima, Kazumasa Yamagishi, Atsushi Goto

**Affiliations:** 1 Department of Preventive Cardiology, National Cerebral and Cardiovascular Center, 5-7-1, Fujishiro-dai, Suita, Osaka 565-8565 Japan; 2 Institute of Cardiovascular and Medical Sciences, University of Glasgow, Glasgow, UK; 3 Division of Hypertension and Nephrology, Department of Medicine, National Cerebral and Cardiovascular Center, Suita, Japan; 4 Department of Public Health Medicine, Faculty of Medicine, and Health Services Research and Development Center, University of Tsukuba, Tsukuba, Japan; 5 Epidemiology and Prevention Group, Center for Public Health Sciences, National Cancer Center, Tokyo, Japan

**Keywords:** Gene and environmental interaction, Hypertension, Lifestyle, Epidemiology, Hypertension guideline

## Abstract

Risk factors for hypertension consist of lifestyle and genetic factors. Family history and twin studies have yielded heritability estimates of BP in the range of 34–67%. The most recent paper of BP GWAS has explained about 20% of the population variation of BP. An overestimation of heritability may have occurred in twin studies due to violations of shared environment assumptions, poor phenotyping practices in control cohorts, failure to account for epistasis, gene-gene and gene-environment interactions, and other non-genetic sources of phenotype modulation that are suspected to lead to underestimations of heritability in GWAS. The recommendations of hypertension guidelines in major countries consist of the following elements: weight reduction, a healthy diet, dietary sodium reduction, increasing physical activity, quitting smoking, and moderate alcohol consumption. The hypertension guidelines are mostly the same for each country or region, beyond race and culture. In this review, we summarize gene-environmental interactions associated with hypertension by describing lifestyle modifications according to the hypertension guidelines. In the era of precision medicine, clinicians who are responsible for hypertension management should consider the gene-environment interactions along with the appropriate lifestyle components toward the prevention and treatment of hypertension. We briefly reviewed the interaction of genetic and environmental factors along the constituent elements of hypertension guidelines, but a sufficient amount of evidence has not yet accumulated, and the results of genetic factors often differed in each study.

Hypertension is the most influential risk factor for cardiovascular disease (CVD) [1]. Recent evidence has suggested that hypertension is also associated with common non-CVD such as dementia and renal dysfunction [2]. Risk factors for hypertension consist of lifestyle and genetic factors. Family history and twin studies have yielded heritability estimates of blood pressure (BP) in the range of 34–67% [3]. The collective effect of all BP loci identified through genome-wide association studies (GWAS) accounted for only ~ 3.5% of BP variability [4]. The most recent paper of BP GWAS has identified 901 SNPs with BP and explained about 20% of the population variation of BP [5]. An overestimation of heritability may have occurred in twin studies due to violations of shared environment assumptions, poor phenotyping practices in control cohorts, failure to account for epistasis, gene-gene (G × G) and gene-environment (G × E) interactions, and other non-genetic sources of phenotype modulation that are suspected to lead to underestimations of heritability in GWAS.

The recommendations of hypertension guidelines in major countries consist of the following elements: weight reduction, a healthy diet (dietary patterns characterized by a high consumption of fruit, vegetables, whole grains, legumes, seeds, nuts, fish, low-fat dairy, and a low consumption of meat and sweets), dietary sodium reduction, increasing physical activity, quitting smoking (including avoiding passive smoking), and moderate alcohol consumption (Table 1) [6–8]. The hypertension guidelines are mostly the same for each country or region, beyond race and culture [9]. In this review, we summarize gene-environmental interactions associated with hypertension by describing lifestyle modifications according to the hypertension guidelines.

**Table 1:** Comparison between three major lifestyle modifications in the hypertension guidelines

	ESH/ESC Guideline 2018 [6]	ACC/AHA Guideline 2017 [7]	JSH Guideline 2014 [8]
Dietary sodium restriction	Salt restriction to < 5 g/day	Optimal goal is < 1500 mg/day, but aim for at least a 1000 mg/day reduction in most adults.	The target of salt reduction is < 6 g/day.
Other dietary changes	Increased consumption of vegetables, fresh fruits, fish, nuts, and unsaturated fatty acids (olive oil); low consumption of red meat; and consumption of low-fat dairy products	A heart-healthy diet, such as the DASH diet, that facilitates achieving a desirable weight is recommended for adults with elevated BP or hypertension. Potassium supplementation, preferably in dietary modification, is recommended for adults with elevated BP or hypertension, unless contraindicated by the presence of CKD or use of drugs that reduce potassium excretion.	Dietary pattern: fruit/vegetable intake should be increased, and cholesterol/saturated fatty acid intake should be reduced. Fish (fish oil) intake should also be increased.
Weight reduction	Body-weight control is indicated to avoid obesity (BMI > 30 kg/m^2^ or waist circumference > 102 cm [men] and > 88 cm [women], as is aiming at healthy BMI (about 20–25 kg/m^2^) and waist circumference (< 94 cm [men] and < 80 cm [women])	Weight loss is recommended to reduce BP in adults with elevated BP or hypertension who are overweight or obese.	The target body mass index is < 25 kg/m^2^. Even when the target is not reached, a significant decrease in blood pressure can be achieved by reducing body weight by approximately 4 kg.
Regular physical activity	Regular aerobic exercise (e.g., at least 30 min of moderate dynamic exercise on 5–7 days/week)	Increased physical activity with a structured exercise program is recommended for adults with elevated BP or hypertension.	Primarily periodic (30 min or longer daily if possible) and aerobic exercise should be practiced.
Smoking cessation	Smoking cessation, supportive care, and referral to smoking cessation programs	Quit cigarette smoking and second-hand smoking.	Smoking cessation should be promoted, and passive smoking must be avoided.
Moderate alcohol consumption	Men: < 14 units/week Women: < 8 units/week Avoid binge drinking	Adult men and women with elevated BP or hypertension who currently consume alcohol should be advised to drink no more than 28 g/day and 24 g/day as ethanol, respectively.	Alcohol intake should be restricted. < 20–30 mL/day in men and < 10–20 mL/day in women as ethanol.

## Gene-sodium interaction

The INTERSALT study indicated an association between overdose salt intake and high blood pressure [10]. The Dietary Approaches to Stop Hypertension (DASH) study showed that sodium intake restrictions from a high level to an intermediate level and from an intermediate to a low level reduced both systolic blood pressure (SBP) and diastolic blood pressure (DBP) [11]. In a pooled analysis of data, lowering sodium intake was shown to be best-targeted at individuals with hypertension who consume high-sodium diets [12]. On the basis of these results, hypertension management guidelines recommend the following: salt intakes of < 5 g/day in Europe [6], < 6 g/day in Japan [8], and sodium intake of < 1500 mg/day (salt intake of < 3. 81 g/day equivalent) in the USA [7].

Salt sensitivity is an increase in BP in response to excessive dietary salt intake, and it is associated with genetic and environmental factors. Salt sensitivity is more frequently observed in hypertensive than normotensive subjects, in colored races than in Caucasians, and in older than in younger subjects [13, 14]. When gene-sodium interactions are studied, the investigations must consider the race and age group of subjects.

A cross-sectional study in Korea indicated that the mutant alleles of *CSK* rs1378942 and *CSK*-*MIR4513* rs3784789 had the strongest protective effects against hypertension in the subjects in the middle group of the 24-h estimated urinary sodium-potassium excretion ratio (Table 2) [15]. In a cross-sectional study in China, Li et al. showed that the interaction for *CLGN* rs2567241 was associated with the sodium intake’s effects on SBP, DBP, and mean blood pressure (MBP), the impact of *UST* rs13211840 on DBP, and the effect of *LOC105369882* rs11104632 on SBP through the examination of an SNP [16]. Also, genome-wide gene-based interactions with sodium identified *MKNK1*, *C2orf80*, *EPHA6*, *SCOC*-*AS1*, *SCOC*, *CLGN*, *MGAT4D*, *ARHGAP42*, *CASP4*, and *LINC01478* which were associated with at least one BP variable. In Chinese Kazakh women, an interaction of *ACE* genotype and salt intake on hypertension was observed [17].

**Table 2:** Review for interaction of gene and salt intake on hypertension

Population	Gene	SNPs/gene length, bp	Chr	Position	Trait		Reference
Korea	LOC101929750	rs7554672	1	219339781	HT	24hUNa, K	15
	MKLN1	rs1643270	7	130826034	HT	24hUK	
	CSK	rs1378942	15	72864420	HT	24hUNa/K	
	CSK-MIR4513	rs3784789	15	72869605	HT		
	TENM4	rs10466739	11	78290369	HT		
Taiwan	GNB3	rs5443	10		HT	Salt intake	22
China	CLGN	rs2567241	4	141542612	SBP, DBP, MBP	Salt intake	16
	LOC105	rs11104632	12	86747816	SBP		
	UST	rs13211840	6	149153883	DBP		
China	MKNK1	46889	1	46795665	SBP	Salt intake	17
	SCOC	39097	4	141484064	SBP, DBP, MBP		
	SCOC-AS1	89668	4	141424329	DBP, MBP		
	CLGN	39210	4	141529056	SBP, DBP, MBP		
	MGAT4D	55004	4	141583978	SBP, DBP, MBP		
	LINC01478	208264	18	40157397	SBP		
	C2orf80	24704	2	208738315	PP		
	EPHA6	429464	3	98641126	PP		
	ARHGAP42	303251	11	100063616	PP		
Japan	NPPA	rs5063	1	11907648	SBP	Salt intake	18
Japan	CYP3A5	rs776746	3		SBP, DBP	24hUNaCl	21
Japan	AGT	T174 M			HT	24hUNa, sodium intake	19
Japan	ADD1	G460 W			SBP	24hUNa, sodium intake	20

*HT* hypertension, *SBP* systolic blood pressure, *DBP* diastolic blood pressure, *MBP* mean blood pressure, *PP* pulse pressure, *24hUNa* 24-h sodium excretion; 24-h potassium excretion; 24-h salt excretion

In a Japanese population, the interaction between salt consumption and *NPPA* rs5063 (Val32Met) showed a significant association with SBP [18]. In a general Japanese population, a high sodium intake strengthened the association of *AGT* T174 M [19] and *ADD1* G460 W (only women) [20] polymorphisms with hypertension and SBP levels, respectively. Another cross-sectional study showed that *CYP3A5* variants might be a determinant of salt sensitivity of BP in Japanese men [21]. A case-control study in Taiwan showed that *GNB3* C825T polymorphism might increase the risk of hypertension among individuals who consumed a high-sodium diet [22]. Adamo et al. reviewed studies of gene-salt interaction [23], but most of those studies might have been subject to error due to their small sample sizes. Studies of gene-environmental interactions require large sample sizes as they involve the grouping of genes and environmental factors.

## Gene-healthy diet interaction

The DASH diet study showed no significant BP lowering in the control group, and the fruits/vegetable group, but SBP and DBP lowering were observed in the DASH diet group [24]. In a meta-analysis of 17 randomized controlled trials, significant reductions of 4.3 mmHg in SBP and 2.4 mmHg in DBP were observed in healthy dietary patterns, including the DASH diet, Nordic diet, and Mediterranean diet, all of which include the high consumption of fruit, vegetables, whole grains, legumes, seeds, nuts, fish, and dairy and a low consumption of meat, sweets, and alcohol [25]. These foods or combinational foods contribute to the prevention of high blood pressure.

A 2-year-randomized intervention trial revealed significant interactions between the Neuropeptide Y (*NPY*) rs16147 SNP and dietary fat intake in relation to changes in SBP and DBP (Table 3) [26]. The gene-diet interactions appeared only in hypertensive patients. During the 2 years of intervention, the subjects with C allele had greater reductions in SBP and DBP in response to a low-fat diet but had greater increases in SBP and DBP in response to a high-fat diet. *NPY* is implicated in the regulation of BP, and *NPY* pathways in the hypothalamus are sensitive to dietary fat. Animal experiments indicated that fat intake and *NPY* activity in the hypothalamus are inversely correlated [27].

**Table 3:** Review for interaction of gene and healthy diet on hypertension

National	gene	SNPs/gene length, bp	Chr	Results	Healthy diet	Reference
USA	NPY	rs16147		SBP, DBP	Dietary fat intake	26
Korea	CYP4F2	433VV		BP change	ω-3 PUFA	28
Japan	COMT	Val158Met	22	higher BP and HT	High-energy intake	30
Spain	NOS3	rs1799983		DBP	Monounsaturated fatty acid	31
					Saturated fatty acid	

See Table 2 footnote

A Korean genome and epidemiology study showed that a higher omega-3 (ω-3) polyunsaturated fatty acid (PUFA) intake was significantly associated with a more pronounced BP decrease over time in subjects with the *CYP4F2* 433VV genotype, although there was no association between ω-6 and ω-3 PUFA intakes, ω-6/ω-3, and changes of BP [28]. A meta-analysis of interventional studies showed that the intake of fish oil caused a decrease in BP in hypertensive patients [29].

In a study of Japanese men, the Met allele of *COMT* Val158Met was associated with higher BP and a higher prevalence of hypertension in the high-energy intake group but not in the low-energy intake group [30]. There was no difference in body mass index (BMI) between the low- and high-energy intake groups. The underlying mechanism of these results remains unclear.

In a Southern European study, there was an interaction between the *NOS3* rs1799983 polymorphism and dietary saturated fatty acid and monounsaturated fatty acid that influenced DBP levels [31]. Martins et al. showed that nitric oxide synthase (NOS) activity was increased in an unsaturated high-fat diet group. The expressions of endothelial NOS (eNOS) and inducible NOS (iNOS) were also increased in the unsaturated high-fat diets group [32]. These changes may be involved in gene-dietary interactions.

## Gene-alcohol interaction

Alcohol consumption is higher among East Asian men compared to Western men, but the consumption of alcohol by Western women is higher than that among East Asian women [33]. Approximately half of East Asians are found to be aldehyde dehydrogenase (ALDH) deficient, which accounts for a phenomenon called the ‘Oriental flushing syndrome.’ ALDH deficiency poses an increased risk of high BP [34].

In a study of middle-aged Finnish men, the apolipoprotein E phenotype significantly influenced the BP increasing effect of alcohol consumption (Table 4) [35]. A cross-sectional study of a Chinese population showed a significant interaction between the *CYP11B2* genotype [36] and DNA methylation (CpG1 methylation) of the *ADD1* gene promoter [37] and alcohol consumption on the risk of hypertension. In addition, the Stanford Asia-Pacific Program for Hypertension and Insulin Resistance (SAPPHIRe) study showed that *ALDH2* genetic variants were associated with progression to hypertension in a prospective Chinese cohort [38]. In a cross-sectional study of 5724 Japanese participants, *ALDH2* rs671 significantly and synergistically influenced the subjects’ drinking behavior and influenced the level of BP independently of the amount of alcohol consumption [39], but not in another study, in a case-control study of 532 Japanese patients, there was no significant interaction between the *ALDH2* genotype and alcohol consumption overall or in Japanese male patients: this study may have had insufficient power to detect the interaction [40].

**Table 4:** Review for interaction of gene and alcohol intake on hypertension

Population	Gene	SNPs/gene length, bp	Chr	Position	Results	Drink	Ancestor	Reference
Finland	APOE				SBP	LHD		35
China	ADD1	rs4961	4		HT	alcohol/w		37
China	CYP11B2				HT	alcohol/w		36
China	ALDH2	rs2238152	12	111776655	HT	LHD		38
Japan	ALDH2	rs671	12		HT	alcohol/w		39
USA	MGC27382-PTGFR	rs648425	1	78659796	SBP	Drinks/w		41
	ESRRG	rs17669622	1	214823444	MAP	Drinks/w		
	RAB4A	rs16849553	1	227403469	MAP	Oz alcohol/w		
	FAM179A	rs13008299	2	29101501	DBP	Drinks/w		
	CRIPT-SOCS5	rs4953404	2	46739646	PP	Days drinks/w, Oz alcohol/w		
	KAT2B	rs9874923	3	20076567	MAP	Drinks/w		
	Intergenic	rs3852160	5	5875647	MAP	Days drinks/w		
	ADCY2	rs4537030	5	7296981	MAP	Drinks/w		
	GLI3	rs7791745	7	42351145	MAP	Drinks/w		
	ZNF716	rs11766519	7	57587798	PP	Days drinks/w		
	SLC16A9	rs10826334	10	61050488	SBP,MAP	Oz alcohol/w		
	SLC16A9	rs10826334	10	61050488	SBP	Drinks/w		
	SLIT1	rs12773465	10	98784049	MAP	Drinks/w		
	SLIT1	rs7902871	10	98799693	DBP	Drinks/w		
	Intergenic	rs7116456	11	23911889	SBP	Drinks/w		
	Intergenic	rs12292796	11	39382675	PP	Drinks/w		
	PDE3A	rs10841530	12	20490379	SBP	Drinks/w		
	KERA-LUM	rs991427	12	89998553	SBP	Oz alcohol/w		
	KERA-LUM	rs4494364	12	90001245	SBP	Drinks/w		
	RNF219-AS1	rs9318552	13	77923788	DBP	Oz alcohol/w		
	CLEC3A	rs2735413	16	76611144	SBP	Drinks/w		
	WFDC1	rs16963349	16	82895735	SBP	Drinks/w		
	FBXO15	rs1943940	18	69856172	DBP,MAP	Drinks/w		
	IGSF5	rs2410182	21	40101946	SBP	Oz alcohol/w		
	IGSF5-PCP4	rs2837253	21	40143126	SBP	Drinks/w		
Multiple	BLK	rs2409784	8	11539347	DBP	CURD	EA,HA	42
	BLK	rs6983727	8	11558303	SBP	LHD	EA	
	BLK	rs6983727	8	11558303	PP	CURD,LHD	EA	
	BLK	rs34190028	8	11559641	SBP	CURD	EA	
	CDH17	rs115888294	8	94105161	PP	CURD	AA	
	CORO2A	rs73655199	9	98145201	PP	CURD	AA	
	ELMOD1	rs139077481	11	107579224	PP	CURD	AA	
	ERCC6	rs4253197	10	49473111	PP	CURD	AA	
	EYS	rs80158983	6	65489746	SBP	CURD	AA	
	FAM167A	rs12156009	8	11427710	SBP	CURD	EA	
	FAM167A	rs13255193	8	11451683	SBP	LHD	EA	
	FAM167A-AS1	rs9969423	8	11398066	SBP	CURD,LHD	EA	
	FTO	rs9928094	16	53765993	PP	CURD	ASA,EA	
	FTO	rs55872725	16	53775211	SBP	CURD	EA	
	FTO	rs7185735	16	53788739	PP	CURD	EA	
	FTO	rs62033406	16	53790314	MAP	CURD	ASA,EA	
	GALNT18	rs10741534	11	11233360	SBP	CURD	AA	
	GATA4	rs3735814	8	11749887	SBP	CURD	EA,HA	
	GATA4	rs36038176	8	11752486	SBP	CURD	EA	
	LINC00208	rs899366	8	11572976	MAP	CURD	EA	
	LINC00208	rs7464263	8	11576667	SBP	LHD	EA	
	LINC00208	rs2244894	8	11591150	PP	CURD	ASA,EA	
	LINC00208	rs1478894	8	11591245	SBP	CURD	EA	
	LINC00208	rs4841569	8	11594668	PP	CURD,LHD	EA	
	LINC00208	rs13249843	8	11601509	DBP	CURD	EA,HA	
	LINC00208	rs17807624	8	11605506	DBP	CURD	EA	
	LINC00208	rs17807624	8	11605506	MAP	LHD	EA	
	LOC102723313	rs13276026	8	10752445	SBP	CURD	EA	
	LOC102723313	rs13276026	8	10752445	DBP,MAP	CURD	EA,HA	
	LOC102724880	rs453301	8	9172877	SBP	CURD	EA	
	LOC102724880	rs453301	8	9172877	DBP	CURD	EA,HA	
	LOC105372045	rs140520944	18	29508647	PP	CURD	AA	
	LOC105372361	rs142673685	19	31669942	PP	CURD	AA	
	LOC105379224	rs2980755	8	8506173	SBP,PP	LHD	EA	
	LOC105379224	rs10092965	8	8515975	DBP	CURD	EA,HA	
	LOC105379224	rs13270194	8	8520592	SBP	CURD	EA	
	LOC105379224	rs7823056	8	8525195	SBP,PP	LHD	AA,EA	
	LOC105379224	rs6995407	8	8527137	PP	CURD	EA	
	LOC105379231	rs6601302	8	9381948	SBP	CURD	EA	
	LOC105379235	rs9650622	8	9946782	DBP	CURD	EA	
	LOC105379235	rs56243511	8	9948185	SBP	CURD	EA	
	LOC105379235	rs656319	8	9956901	SBP,MAP	LHD	EA	
	LOC105379242	rs13280442	8	11610048	SBP,MAP	CURD,LHD	EA	
	LOC105379242	rs13250871	8	11610254	PP	CURD,LHD	EA	
	LOC107986913	rs2979172	8	8452998	PP	LHD	EA	
	LOC107986913	rs2921064	8	8459127	PP	CURD	EA	
	LOC107986913	rs2979181	8	8465578	SBP	CURD,LHD	EA	
	LOC157273	rs10503387	8	9293015	SBP	CURD	AA,EA	
	LOC157273	rs11781008	8	9295729	DBP	CURD	EA,HA	
	LOC157273	rs11774915	8	9331252	SBP	CURD	EA	
	MIR124–1	rs483916	8	9936091	SBP,DBP,PP	CURD	EA	
	MIR124–1	rs615632	8	9938811	SBP	LHD	EA	
	MIR4286	rs7814795	8	10661775	SBP	CURD,LHD	EA	
	MIR4286	rs7814795	8	10661775	MAP	CURD	EA	
	MIR4286	rs28680211	8	10661935	MAP	LHD	EA	
	MSRA	rs2062331	8	10122482	DBP	CURD	EA	
	MSRA	rs11993089	8	10152442	PP	CURD	EA	
	MSRA	rs34919878	8	10241994	DBP	CURD	EA,HA	
	MSRA	rs4841294	8	10247558	SBP	LHD	AA,EA	
	MSRA	rs17693945	8	10248500	MAP	LHD	AA,EA	
	MSRA	rs7832708	8	10332530	SBP	LHD	EA	
	MSRA	rs11786677	8	10406750	SBP	CURD	EA	
	PINX1	rs4551304	8	10807559	DBP,MAP	CURD	EA,HA	
	PINX1	rs7814757	8	10817678	SBP	CURD	EA	
	RP1L1	rs4841409	8	10658864	SBP	CURD	EA	
	RP1L1	rs4841409	8	10658864	MAP	CURD,LHD	EA	
	RP1L1	rs10096777	8	10660990	SBP	LHD	EA	
	TACC2	rs11200509	10	122256927	PP	LHD	AA	
	TARID	rs76987554	6	133759717	SBP	CURD	AA	
	TNKS	rs4383974	8	9761838	SBP	CURD	AA,EA	
	TNKS	rs35231275	8	9762399	PP	CURD	EA	
	TNKS	rs9286060	8	9795635	DBP	CURD	AA,EA	
	TNKS	rs1976671	8	9822124	SBP	CURD	EA	
	TNKS	rs55868514	8	9822890	DBP	CURD	EA	
	UNC5D	rs79505281	8	35841899	PP	CURD	AA	
	XKR6	rs4841465	8	10962344	SBP	CURD,LHD	EA	
	XKR6	rs9969436	8	10985149	MAP	LHD	AA,EA	

*HT* hypertension, *SBP* systolic blood pressure, *DBP* diastolic blood pressure, *MBP* mean blood pressure, *PP* pulse pressure, *CURD* current drinker (yes/no), *LHD* light (1 ± 7 drinks/week) drinking; Ancestry, *EA* European ancestry, *AA* African American ancestry, *ASA* Asian American ancestry, *HA* Hispanic ancestry

A genome-wide analysis of the effect of SNP-alcohol interactions on BP traits showed 1 significant and 20 suggestive BP loci by exploiting gene-alcohol interactions in a study from the Framingham SNP Health Association Resource [41]. The CHARGE Gene-Lifestyle Interactions Working Group has systematically shown the gene-alcohol interaction on BP in a recent and extensive meta-analysis across multiple ancestries, conducting a large two-stage investigation incorporating joint testing of main genetic effects and single nucleotide variant (SNV)-alcohol consumption interactions [42]. The study identified and replicated 54 BP loci in European ancestry and multi-ancestry meta-analyses.

## Gene-smoking interaction

According to the Global Burden of Disease Study 2015, central and eastern Europe and southeast Asia had a higher prevalence of smoking than the global average for men, and western and central Europe had a higher prevalence of smoking than the global average for women [43]. The population-attributable fractions of coronary heart disease caused by smoking among men and women were higher in the East Asian region than in the Western Pacific region [44].

In a rural Chinese population, the cigarette smoking index and *ACE* gene showed a low exposure-gene effect on essential hypertension with interaction indices (Table 5) [45]. In an eastern Chinese Han population, gene-environment interactions between rs1126742 and smoking were associated with an increased risk of essential hypertension [46]. A case-control study showed the association of *KCNJ11* gene polymorphisms and BP response to the antihypertensive drug irbesartan in non-smoking Chinese hypertensive patients [47]. As a genome-wide study, the Framingham Heart Study identified 7 significant and 21 suggestive BP loci by gene-smoking interactions in an analysis of 6889 participants [48].

**Table 5:** Review for interaction of gene and smoking on hypertension

Population	Gene	SNPs/gene length, bp	Chr	Position	Results	Smoking	Reference
China	ACE	I/D			EH	Smoking	45
China	KCNJ11				HT	Non-smoking	46
China	CYP4A11	rs1126742	1		EH	Smoking	47
USA	LOC729336	rs11589828	1	230735895	SBP	Pack-years	48
	LRP1B	rs1033284	2	141638258	SBP	Pack-years	
	LRP2	rs2268365	2	169802415	SBP	Pack-years	
	FLJ45964	rs11679072	2	240109156	SBP	Pack-years	
	CNTN4	rs9878978	3	2460969	SBP	Pack-years	
	MECOM	rs12634933	3	170512673	SBP	Pack-years	
	PRKG2	rs17484474	4	82345145	SBP	Pack-years	
	GYPA-KRT18P51	rs6537278	4	145477389	SBP	Pack-years	
	RPS6KA2	rs4710117	6	167184091	SBP	Pack-years	
	PPP1R3A-FOXP2	rs12705959	7	113785482	SBP	CPD	
	COLEC10-MAL2	rs6989684	8	120212220	SBP	Pack-years	
	TRAPPC9	rs7823724	8	141473511	SBP	Pack-years	
	ADARB2	rs6560743	10	1627136	SBP	Pack-years	
	OPCML	rs7104871	11	132544409	SBP	Pack-years	
	CACNA2D4	rs2286379	12	1772425	SBP	Pack-years	
	SACS-TNFRSF19	rs2297585	13	22942344	SBP	Pack-years	
	FRY	rs9533282	13	31525648	SBP	Pack-years	
	GPC5-GPC6	rs9561252	13	92527286	SBP	CPD	
	LOC730007	rs8010717	14	79480194	SBP	CPD	
	NRXN3	rs8010717	14	79480194	SBP	Pack-years, smoking	
	HERC2P6	rs937741	15	21198852	SBP	CPD	
	CYB5B	rs12149862	16	68054704	SBP	Pack-years	
	ZSWIM7	rs7211756	17	15840400	SBP	Pack-years	
	CDH19-DSEL	rs7234531	18	62721365	SBP	Pack-years	
	MN1	rs133980	22	26352728	SBP	CPD, Pack-years	
	LOC200810	rs7615952	3	127132093	DBP	Pack-years	
	GRB10	rs10275663	7	50765179	DBP	CPD	
African American	NEDD8	rs11158609	14	24688814	SBP	Smoking	49
	TTYH2	rs8078051	17	72251240	SBP	Smoking	

*HT* hypertension, *SBP* systolic blood pressure, *DBP* diastolic blood pressure, *MBP* mean blood pressure, *PP* pulse pressure, *CPD* cigarettes per day

The further genome-wide research was proposed to examine African American participants in the Hypertension Genetic Epidemiology Network (HyperGEN) research, and testing the association in African American participants from the Genetic Epidemiology Network of Arteriopathy (GENOA) study [49]. The results suggested that *NEDD*8 rs11158609 and *TTYH2* rs8078051 were associated with SBP including the genetic interaction with cigarette smoking, although these two SNPs were not associated with SBP in a main genetic effect model.

## Gene-obesity interaction

Globally, the prevalence of overweight or obesity for adults increased from 28.8% and 29.8% in 1980 to 36.9% and 38.0% in 2013 for men and women, respectively, which were observed in both developed and developing countries [50]. The prevalence of overweight and obesity is rising among children and adolescents in developing countries as well, rising from 8.1% and 8.4% in 1980 to 12.9% and 13.4% in 2013 for boys and girls, respectively. A meta-analysis of 25 studies has estimated that as body weight decreased by 1 kg, SBP and DBP decreased by − 1.05 mmHg and − 0.92 mmHg, respectively [51]. Therefore, weight loss for obese people is an essential factor in lowering BP.

The Atherosclerosis Risk in Communities Study showed a significant interaction among the *GNB3* C825T polymorphism, obesity status, and physical activity in predicting hypertension in African American subjects, and those who were both obese and had a low activity level with T allele were 2.7 times more likely to be hypertensive compared to non-obese, active C homozygotes [52].

The representative SNPs related to BMI are those in *FTO* and *MC4-R* loci. SNPs in *FTO* were associated with hypertension in different ethnic groups [53]. The Pima Indians in Arizona have the highest prevalence of obesity in the world, but a relatively low prevalence of hypertension and atherosclerotic disease [54]. The lack of increase in muscle sympathetic nerve activity with increasing adiposity and insulinemia in Pima Indians may explain this in part [55], but the reason why this population has a low tendency for hypertension despite the high prevalence of obesity and hyperinsulinemia are not yet known.

## Gene-physical activity interaction

A meta-analysis that included 13 prospective studies suggested that there was an inverse dose-response association between levels of recreational physical activity and risk of hypertension [56]. A recent systematic review and meta-analysis of randomized control trials with a meta-regression of potential effect modifiers revealed that exercise was associated with a reduction in SBP of − 4.40 mmHg and in DBP of − 4.17 mmHg at 3–6 months after the intervention began [57]. Potential reasons for the association between physical activity and BP decreases are as follows. First, physical activity helps maintain appropriate body weight. Second, exercise decreases total peripheral resistance [58]. Physical activity has also been shown to improve insulin sensitivity [59], which increases high blood pressure via its effect in increasing sodium reabsorption and sympathetic nervous system activity [60]. An exercise habit can also help improve one’s other lifestyle habits. Individuals who exercise every day tend to focus on improving their lifestyle in other aspects of their daily lives.

In a cross-sectional study of African American women, *SLC4A5* rs1017783 had a significant interaction with A allele and AA genotype by physical activity on SBP and DBP, respectively. In addition, *SLC4A5* rs6731545 had a significant interaction with GA genotype by physical activity on both SBP and DBP. A study of Chinese children showed that interactions between a genetic risk score including *ATP2B1* rs17249754, fibroblast growth factor 5 (*FGF5*) rs16998073 polymorphisms, and physical activity play important roles in the regulation of BP and the development of hypertension [61]. *ATP2B1* is expressed in the vascular endothelium and regulates the homeostasis of cellular calcium levels, which is important in controlling the contraction and dilation of vascular smooth muscles [62]. The most commonly cited effect of *FGF*-*5* is to promote angiogenesis in the heart. *FGF*-*5* acts as an autocrine/paracrine mechanism of cardiac cell growth and as a cytoprotective mechanism against irreversible ischemic damage [63]. *FGF*-*5* rs16998073 polymorphisms were significantly associated with hypertension risk in East Asians [64]. However, no evidence supports a role for this gene in the pathogenesis of hypertension.

## Perspectives

In the era of precision medicine, clinicians who are responsible for hypertension management should consider the gene-environment interactions along with the appropriate lifestyle components toward the prevention and treatment of hypertension. The effects and contributions of other confounding and interaction factors such as race, age, other lifestyle habits (e.g., lack of sleep [65] and bathing [66]), and environmental factors (e.g., weather conditions [67] and air pollution [68]), stress [69], and social factors [70] must also be determined comprehensively.

We briefly reviewed the interaction of genetic and environmental factors along the constituent elements of hypertension guidelines, but a sufficient amount of evidence has not yet accumulated, and the results of genetic factors often differed in each study. The following requirements should be considered in future studies: (1) set of the reproducible environmental factor with simple and easy way; (2) consider the subjects’ race, gender, and age; (3) select research subjects so that bias is as small as possible; (4) use a risk score of the target disease including a simple dietary intake and physical activity questionnaire and examines genetic factors to improve the risk model; and (5) effectively provide hypertension management with precision medicine based on the components of appropriate lifestyle interventions in hypertension prevention guidelines for a cardiovascular disease model with the specific gene-environmental factors being studied.

The Genetic Epidemiology Network of Salt Sensitivity (The GenSalt) Study obtained novel implications regarding the association between BP responses to dietary sodium and potassium and hypertension and identifying an inverse relation between a BP genetic risk score and salt and potassium sensitivity of BP [71]. The UK Biobank data recently revealed 107 validated loci for BP, in a study that showed that BP which is 9–10 mmHg higher with an over twofold higher risk of hypertension (in a comparison of the top and bottom quintiles of the BP genetic risk score distribution) has potential clinical and public health implications [72]. Although the extent to which each gene contributes to BP is small, by incorporating the concept of a genetic risk score, the contribution of blood pressure has been shown by many GWAS. BP research will continue to contribute to future preventive medicine.

## Conclusion

We summarize gene-environmental interactions associated with hypertension by describing common lifestyle modifications according to the recommendations of hypertension guidelines in major countries which consist of the following elements: weight reduction, a healthy diet, dietary sodium reduction, increasing physical activity, quitting smoking, and moderate alcohol consumption. We briefly reviewed the interaction of genetic and environmental factors along the constituent elements of hypertension guidelines, but a sufficient amount of evidence has not yet accumulated, and the results of genetic factors often differed in each study.

## Abbreviations

ALDH: Aldehyde dehydrogenase
BMI: Body mass index
BP: Blood pressure
CHARGE: Cohorts for Heart and Aging Research in Genetic Epidemiology
CVD: Cardiovascular disease
DASH: Dietary Approaches to Stop Hypertension
DBP: Diastolic blood pressure
eNOS: Endothelial nitric oxide synthase
GENOA: Genetic Epidemiology Network of Arteriopathy
GenSalt: Genetic Epidemiology Network of Salt Sensitivity
GWAS: Genome-wide association studies
HyperGEN: Hypertension Genetic Epidemiology Network
iNOS: Inducible nitric oxide synthase
INTERSALT: International Cooperative Study on Salt, Other Factors, and Blood Pressure
MBP: Mean blood pressure
NOS: Nitric oxide synthase
PUFA: Polyunsaturated fatty acid
SAPPHIRe: Stanford Asia-Pacific Program for Hypertension and Insulin Resistance
SBP: Systolic blood pressure
SNV: Single-nucleotide variant
